# Beyond the Limits of Human Muscle Hypertrophy: A Case Study of a Mr. Olympia Competitor Bodybuilder

**DOI:** 10.1519/JSC.0000000000005519

**Published:** 2026-05-12

**Authors:** Giuseppe Rosaci, Sara Biban, Sandro Bartolomei

**Affiliations:** Department for Life Quality Studies, University of Bologna, Bologna, Italy

**Keywords:** muscle thickness, echo intensity, muscle quality, bodybuilding, ultrasound

## Abstract

Rosaci, G, Biban, S, and Bartolomei, S. Beyond the limits of human muscle hypertrophy: A case study of a Mr. Olympia competitor bodybuilder. *J Strength Cond Res* 40(10): 1253–1258, 2026—This study aimed to investigate changes in body composition and muscle architecture (muscle thickness [MT] and corrected echo intensity [cEI]) between the bulking and the cutting phase of a professional bodybuilder's preparation (age: 38 years; body mass: 128.7 kg; height: 176 cm). MT and cEI of 9 muscles (elbow flexors, triceps brachii, pectoralis major, upper trapezius, vastus medialis/lateralis, biceps femoris, medial/lateral gastrocnemius) were calculated by B-mode ultrasound images. In addition, training and food logs were analyzed, and body composition was estimated by skinfold measurements at the same timepoints. Caloric intake decreased from ∼5,620 kcal·d^−1^ in the bulking phase to ∼3,140 kcal·d^−1^ (−44%) in the cutting phase, while the resistance training program remained largely unchanged. A loss of 6.4 kg was registered in body mass, while a reduction of 0.7 mm only was recorded in the sum of the 3 skinfolds considered. Consistently, all the muscles showed a reduction in MT, particularly evident in the upper trapezius (−28.4%) and in the gastrocnemius medialis (−12.8%). On the contrary, increases in cEI were registered in almost all sites (+28.2% and +30.4% in elbow flexors and biceps femoris, respectively). The increase in cEI registered in the cutting phase, without an increase in fat mass, may be related to a reduction of muscle glycogen. The athlete's subcutaneous fat, together with the combination of cEI and MT, should be monitored during the preparation of an elite bodybuilder to better understand the responses of the main muscles to the nutritional and training strategies adopted.

## Introduction

In bodybuilding contests, athletes pose on stage and are judged based on their aesthetic appearance determined by muscle mass, symmetry, body shape, and muscle definition (3). Athletes are requested to adhere to specific parameters and rules according to the categories in which they compete (e.g., Open, 212, Classic Physique, Men's Physique). The Open category is characterized by an extreme muscle development and definition, whereas other categories require a specific aesthetic or include body mass limitations. Competitions are organized under different federations and levels, with the International Federation of Bodybuilding & Fitness (IFBB) Professional League organizing the most prestigious competition: the Mr. Olympia. Athletes competing in the IFBB Professional League contests are judged according to the highest international standards and are not asked to undergo antidoping controls. To reach their peak physical condition on stage, athletes use different training and nutritional strategies during their preparation, typically including a bulking phase (off-season) and a cutting phase (in-season). These strategies are managed in relation to the athlete's category and to the competitive standards of the federation.

Numerous studies have investigated the athletes' anthropometry (36), body composition (8), nutritional and training strategies (3,12), and muscle architecture (25) of bodybuilders. However, most of these studies did not include repeated measures performed across the different phases of the preparation, and only few studies considered elite athletes (3). Moreno et al. compared body composition and muscle thickness (MT) in 7 IFBB Pro League bodybuilders, and confirmed that the highest skeletal muscle mass index, MTs, and girths were found among the athletes competing in the Open category compared with the other categories. However, these authors did not consider the athlete's training phase in which the assessments were conducted and no parameters of muscle quality, such as echo intensity (EI), were calculated from ultrasound images (25). Bodybuilders aim to achieve a highly muscular physique with a minimal level of body fat, and EI may represent a valuable, noninvasive parameter for the assessment of muscle quality. The measurement of the EI indeed is widely used among athletes of different disciplines and high correlations have been reported between this parameter and strength and power performances. In particular, a low EI (darker images) have been associated with a higher muscle quality, homogeneity, and contractile efficiency (35) and to high strength and power performance capabilities, such as sprint (5,6), peak power output (10), and maximum strength (26). Parameters such as the amount of fibrous tissue within the muscle (33) and the content of muscle glycogen and intramuscular fat (24) are known to influence values of muscle EI. These factors may be influenced by sex, body composition, diet (e.g., muscle glycogen) (16) and by acute or chronic training interventions (37). Reductions in the EI (indicating an improvement in muscle quality) have been reported in the rectus femoris following resistance training programs in both healthy young men (17) and older adults (23).

To the best of our knowledge, no studies to date compared body composition and muscle architecture between the off-season (bulking) and the in-season (cutting) phases in elite bodybuilders competing in the Open category and qualified for the IFBB Pro League Mr. Olympia (34). Therefore, the aim of this study was to analyze these changes during the different preparation phases of a professional bodybuilder and to analyze his food log and training program. Another aim of the present investigation was to assess extremely hypertrophic muscles through B-mode ultrasound and to analyze their properties and architectures. The authors hypothesized that a reduction of both fat and muscle mass, and significant changes in EI and MT may occur in the cutting compared with the bulking phase.

## Methods

### Experimental Approach to the Problem

**Figure 1.**
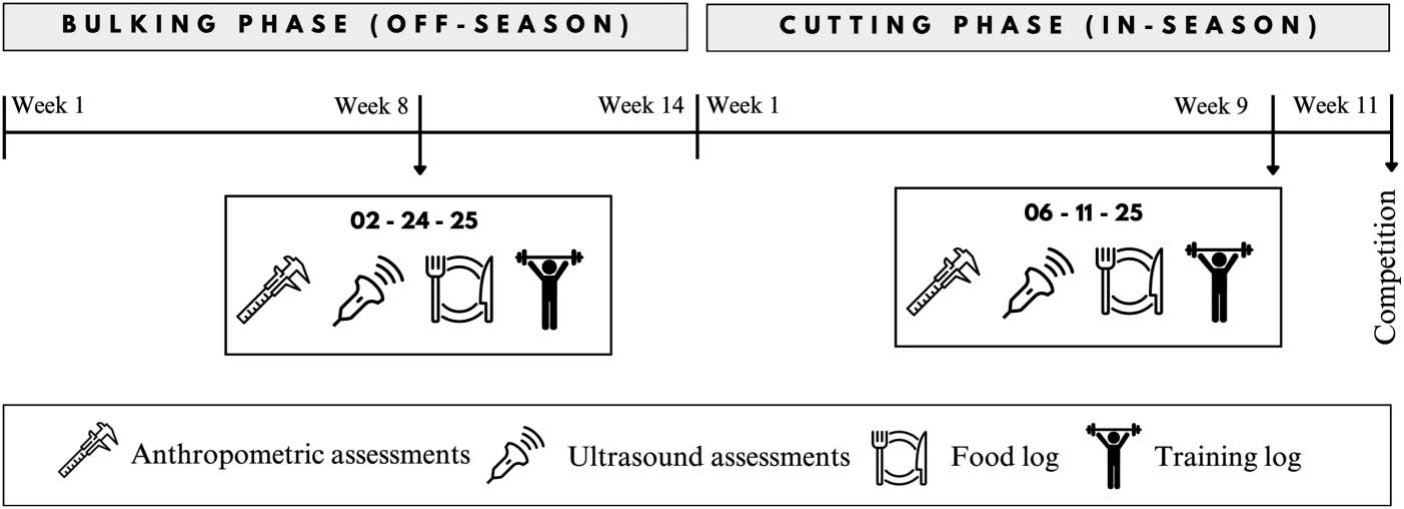
Experiment design of the study.

The experimental design of the case study is reported in Figure 1. The subject was assessed for anthropometric measures (body mass, girths, skinfolds) and muscle architecture in 2 different phases of the preparation: during the bulking phase (February 2nd, 2025; approximately at week 8 of the 14-week phase) and during the cutting phase (June 11th, 2025; approximately at week 9 of the 11-week phase), 10 days before the 2025 IFBB Huanji China Pro League competition. The subject was asked to provide his food log (including supplements) and a typical week of the bulking and the cutting phase was analyzed. In addition, the subjects provided the training programs that were followed in the bulking and in the cutting phase.

### Subject

The subject of the present case study (38 y; 176.0 cm; 128.7 kg; 25 years of training experience) was a professional bodybuilder [Tier 5: world class (24)], who competed in 3 different editions of the IFBB Pro League Mr. Olympia contest in the Open category (Best ranking: 12th position). The subject signed an informed consent form, and the study was approved by the local committee of bioethics (n. 0070230, February 29, 2024). In addition, the athlete provided written informed consent for the use of his image in this study. The subject was asked to abstain from intense physical activity in the 48 hours before the assessment days.

### Dietary Intake and Training Log

During both phases, the subject was under the guidance of his Bodybuilding coach that tailored the training program and the diet and monitored his progress. The subject was asked to provide his food log (including supplements), and a typical week of the bulking and of the cutting phase was analyzed. Thus, daily total Kcal, macronutrients, and water were calculated and reported as average. In addition, the training programs (sessions × week and sets × repetitions) of both bulking and cutting phases, were provided by the athlete. The eventual use of performance enhancing drugs by the athlete, competing in IFBB Pro League contests, in which antidoping tests are not performed, was not considered, nor discussed, in this study.

### Anthropometric Measurements and Body Composition

Athlete's body mass and height were assessed by a scale (K-Deltas, Kinvent Physio, Montpellier, France) and a portable stadiometer (GPM, Zurich, Switzerland), respectively. Three body-girths (flexed and tensed arm, thigh and calf) were measured using a flexible tape. Flexed and tensed arm girths were assessed during the maximum contraction at the point of maximum girth with the arm raised anteriorly and the elbow flexed at 90° (29). Thigh girth was measured between the midpoint of the inguinal crease and the proximal edge of the patella while calf girth was measured at the widest point with a knee angle of 90° (29). All the anthropometric measurements were taken on the right side of the body. Each anthropometric assessment was repeated 3 times and, according to previous literature, the intraoperator measurements error was set <1% (31). In addition, body fat percentage (% body fat) was assessed using the following 3-skinfold equation: % body fat = 8.997 + 0.24658 × (SUM 3 SKF) − 6.343 × (sex) − 1.998 × (race), where SUM 3 SKF was the sum of triceps brachii, abdominal and thigh skinfolds in millimeters with the sex coded as 0 = women and 1 = men and race coded as 0 = White and 1 = Black (11). The triceps brachii skinfold was assessed on the posterior surface of the arm, at the mid-line between the most lateral part of the acromial border and the lateral border of the head of the radius (29). The abdominal skinfold was assessed at 3 cm on the right and 1 cm below the belly button (29). Thigh skinfold was taken at the midpoint between the inguinal ligament and the superior border of the patella (29). Each body composition variable was measured 3 times, and the mean value was reported for further analysis. The researcher's skinfold measurements showed a reliability of Intraclass Correlation Coefficient (ICC) = 0.97 (*SEM* = 0.4 mm). Fat-free mass was then calculated by subtracting the fat mass from the body mass (32). Because the equation used to estimate the percentage of body fat (11) was not specifically validated for top-level body builders, each skinfold and the sum of the 3 skinfolds were also reported.

### Ultrasound Measurements

During the 2 visits, skeletal muscle ultrasounds images were obtained using a 5–12-MHz linear probe (Vscan Air, GE HealthCare, Milan, Italy) placed on the skin surface without depressing the dermal layer (B-mode; gain = 50 dB; image depth = 5 cm). The probe was coated with transmission gel to optimize the spatial resolution. Before the image acquisition, the subject was asked to lie down on a physiotherapy table for at least 15 minutes. MT of 4 upper-body muscles (pectoralis major, upper trapezius, elbow flexors, and triceps brachii) and 5 lower-body muscles (vastus medialis, vastus lateralis, biceps femoris, and medial and lateral gastrocnemius) was assessed. The pectoralis major was measured between the third and fourth ribs below the midpoint of the clavicle (2). The upper trapezius was assessed at the midpoint of the muscle belly between T1 and the posterior acromial edge, where muscle borders were parallel (30). The measurements of the elbow flexors and triceps brachii were collected at 60% distal between the lateral epicondyle of the humerus and the acromial process of the scapula (25). The vastus medialis was measured at the proximal border of the patella (38) while the vastus lateralis along the longitudinal distance, midway between the lateral condyle of the tibia, and the most prominent point of the great trochanter, with a knee flexion of 10° (9). The medial gastrocnemius was measured at 30% of the tibial length, defined as the distance from the popliteal crease to the midpoint of the medial malleolus (22). The lateral gastrocnemius was assessed at 30% proximal between the lateral malleolus of the fibula and the lateral condyle of the tibia (1). For each image, the MT was measured with a single perpendicular line from the superficial aponeurosis to the deep aponeurosis (28). All the measurements were taken by the same investigator (ICC: 0.95; *SEM* = 1.05 mm). Subcutaneous fat thickness (SF) was quantified as the distance between the skin–muscle interface and the superior border of the muscle's aponeurosis (33). The images were imported in a specific software (ImageJ, version 1.53k, National Institutes of Health) where the EI was calculated in the largest possible region of interest, manually selected using the polygon selection tool (21). The raw values of EI were corrected for the subcutaneous fat thickness using the following equation (27):

$$\text{Corrected}\;\;\text{echo}\;\text{intensity}\;\left(\text{cEI}\right)=\text{raw}\;\text{EI}-\left(5.0054\times {\text{SF}}^{2}\right)+\left(\text{SF}\times 38.30836\right)$$

The average of all corrected echo intensity (cEI) values of the different muscles was used to identify a general condition of muscle quality of the athlete.

### Statistical Analyses

Descriptive statistics were reported as means and standard deviations. In addition, percentage differences between the bulking phase (off-season) and the cutting phase (in-season) were calculated for dietary intakes, anthropometric, body composition, and ultrasound measurements.

## Results

### Dietary Intake and Training Log

The subject's average energy intakes during both bulking and cutting phases are summarized in Table 1. During the cutting phase, the athlete's total energy intake was lower by 44% compared with the bulking phase. Carbohydrates intake decreased by 62%, while absolute protein intake remained unchanged. Similarly, fat intake decreased by 40%, while the water intake increased by 25% in the cutting phase compared with the bulking phase. Furthermore, the same supplements were used in both phases.

**Table 1:** Dietary intake (average ±SD) during bulking (off-season) and cutting (in-season) phases of the preparation.

Parameter	Off-season	In-season
Total Kcal	5.620 ± 122 kcal·d^−1^	3.140 ± 93 kcal·d^−1^
Carbohydrates	850 ± 51 g·d^−1^ (∼6.6 g·kg^−1^)	320 ± 30 g·d^−1^ (∼2.6 g·kg^−1^)
Proteins	330 ± 23 g·d^−1^ (∼2.5 g·kg^−1^)	330 ± 18 g·d^−1^ (∼2.7 g·kg^−1^)
Fat	100 ± 14 g·d^−1^ (∼0.78 g·kg^−1^)	60 ± 12 g·d^−1^ (∼0.49 g·kg^−1^)
Water	∼4 L·d^−1^	∼5 L·d^−1^
Supplements	Probiotics; 10g of creatine (following every training session); 20g of essential amino acids (during every training session); Omega 3; vitamins (C, D3, E, B-complex); 1200 mg of N-acetylcysteine (daily); enzymes; monacolin-K.	

The training programs of both bulking and cutting phases are summarized in Table 2. In both phases, each exercise was preceded by at least 2 warm-up sets to reach the appropriate load that ensured the targeted number of repetition (6–8 or 12–15). In addition, all the exercises performed during the training sessions were performed with maximum effort, and each set ended when technical failure occurred. Although some variations may occur in the exercise included in the training program, the structure of the weekly plan and of each workout in number of sets, repetitions, and training sessions remained consistent throughout the study period.

**Table 2:** Training program for the bulking (off-season) and cutting (in-season) phases of the preparation.

Days	Muscles	Number of exercises	Volume	Repetitions
Both bulking and cutting phases				
Monday	Quads	4 exercises	3 sets for exercise (12 sets in tot)	First 2 exercises: range 6–8 reps
				Last 2 exercises: range 12–15 reps
Tuesday	Deltoids	4 exercises	3 sets for exercise (12 sets in tot)	First 2 exercises: range 6–8 reps
				Last 2 exercises: range 12–15 reps
	Triceps	3 exercises	3 sets for exercise (9 sets in tot)	Range 12–15 reps
Wednesday	Back	5 exercises	3 sets for exercise (15 sets in tot)	First 3 exercises: range 6–8 reps
				Last 2 exercises: range 12–15 reps
Thursday	Rest day			
Friday	Hamstrings	2 exercises	4 sets for exercise (8 sets in tot)	Range 12–15 reps
	Biceps	3 exercises	3 sets for exercise (9 sets in tot)	
Saturday	Pectoralis	4 exercises	3 sets for exercise (12 sets in tot)	First 2 exercises: range 6–8 reps
				Last 2 exercises: range 12–15 reps
Sunday	Rest day			
Cutting phase only				
1D/W	Cardio training: 30 min of fast walking			

*Reps = repetitions; 1D/W = 1 day for week.

### Anthropometric and Body Composition

All the anthropometric and body composition data are summarized in Table 3. Body mass and fat-free mass decreased by 4.45 and 4.72% from the bulking to the cutting phase, respectively. Average girth measurements showed reduction by 1.15% (−0.44% in flexed and tensed arm girth, −2.78% in thigh girth, and −0.22% in calf girth). The SUM 3 SKF decreased by 0.7 mm from the bulking to the cutting phase. In addition, the % body fat decreased approximately by 0.2% (0.7 kg).

**Table 3:** Anthropometric and body composition data.*

Measurements	BP	CT
Anthropometric data		
Body mass (kg)	128.7	122.3
Flexed and tensed arm (cm)	52.0	51.8
Thigh (cm)	75.5	73.4
Calf (cm)	44.5	44.4
Body composition data		
SUM 3SKF (mm)	13.9	13.2
Mean abdominal skinfold (mm)	5.3 ± 0.12	5.0 ± 0.15
Mean tricipital skinfold (mm)	3.9 ± 0.17	3.7 ± 0.10
Mean thigh skinfold	4.7 ± 0.12	4.5 ± 0.13
% Body fat (%)	6.1	5.9
Fat-free mass (kg)	120.8	115.1

*SUM 3SKF = sum of 3 skinfolds (triceps brachii, abdominal, thigh); BP = bulking phase (off-season); CT = cutting phase (in-season).

### Ultrasound Assessments

**Figure 2.**
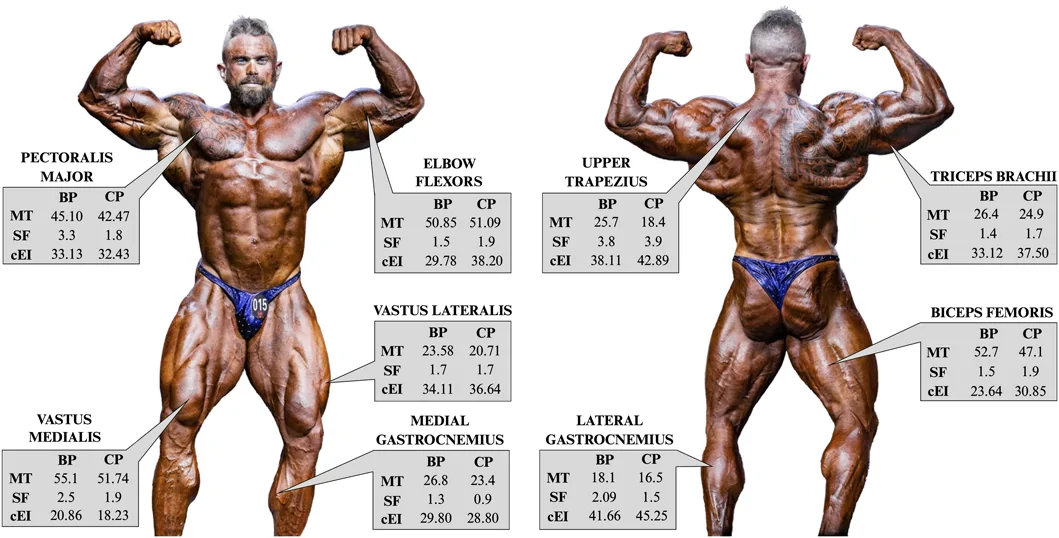
Ultrasound assessments obtained in bulking phase (BP) and cutting phase (CP). Image represents the athlete form in competition, 10 days following the measurements acquisition. MT = muscle thickness (mm); SF = subcutaneous fat (mm); cEI = corrected echo-intensity (au).

**Figure 3.**
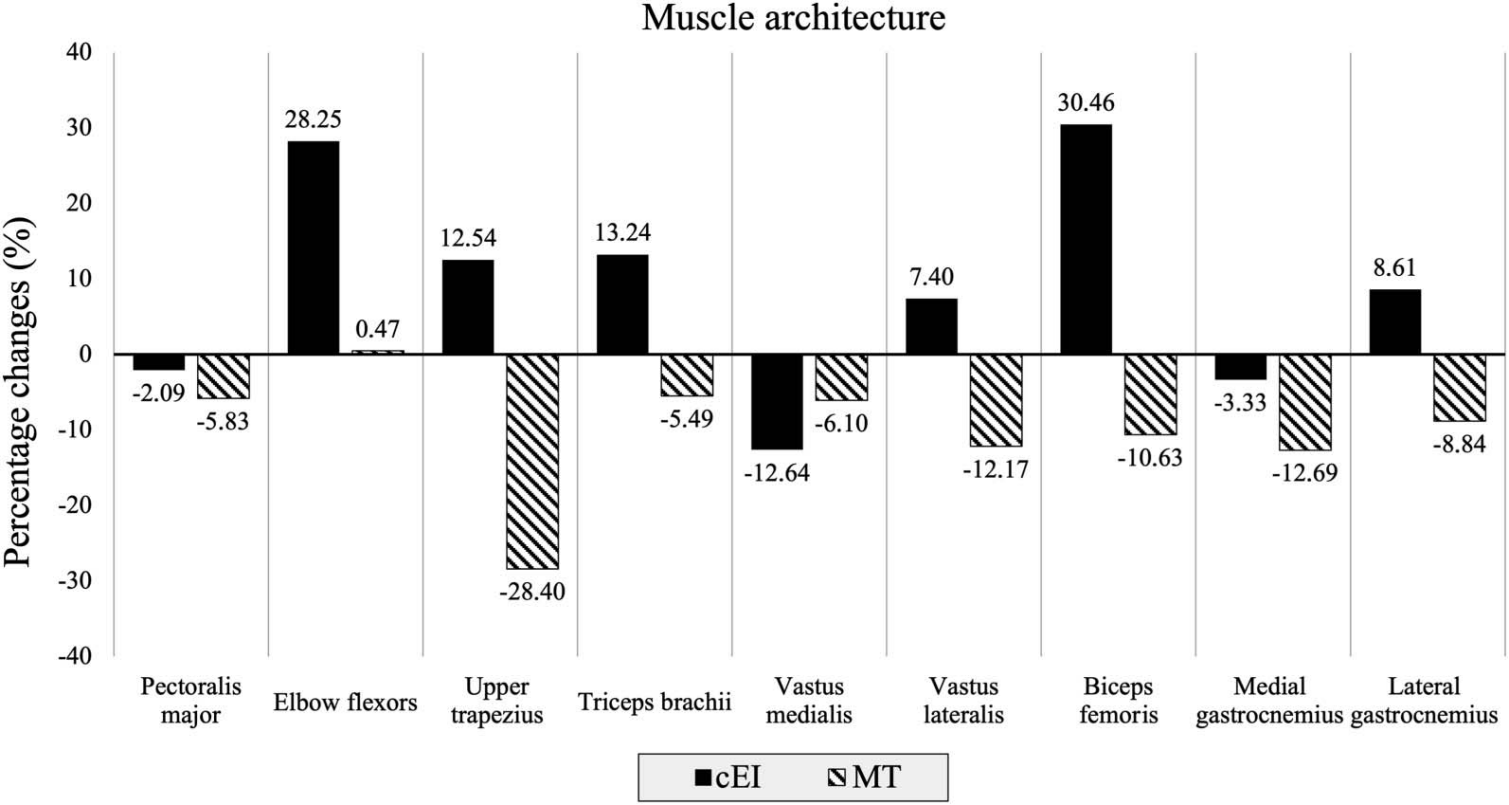
Percentage changes in muscle thickness (MT) and corrected echo intensity (cEI) values between the bulking (off-season) and cutting phases (in-season).

All the MT and cEI values obtained in bulking and cutting phase are reported in Figure 2. The average of MTs decreased from 36.03 ± 14.59 mm (bulking phase) to 32.92 ± 14.83 mm (−8.62%) (cutting phase). However, the most pronounced reductions were observed in the cutting phase for the upper trapezius (−28.4%), gastrocnemius medialis (−12.7%), vastus lateralis (−12.2%), and biceps femoris (−10.6%). The average SF decreased from 2.12 ± 0.9 mm to 1.91 ± 0.81 mm (−5.67%) while mean cEI increased from 31.58 to 34.53 a.u (+9.16%), indicating a generalized rise of this parameter in the different muscle groups in the cutting phase. The most pronounced increases in cEI were observed in the cutting phase on the biceps femoris (+30.5%), elbow flexors (+28.3%), triceps brachii (+13.2%), and upper trapezius (+12.5%). All the percentage changes in MT and cEI values between the bulking phase and the cutting phase are reported in Figure 3.

## Discussion

This study aimed to analyze the changes in anthropometric parameters, body composition, and muscle architecture between the off-season (bulking) and in-season (cutting) phases of an elite bodybuilder preparing for an international IFBB Pro League Open category competition. As expected, the results indicated a decrease in body mass (−4.45%) and MT (−8.62%) in the cutting phase compared with the bulking phase. These results are consistent with previous researches conducted on bodybuilders, in which a reduction in body mass was reported during the cutting phase as a consequence of energy restriction, mainly obtained by a large reduction in carbohydrates intake (4). Interestingly, this occurred despite the daily protein intake was maintained within the upper limit of current recommendations for athletes in hypocaloric conditions (2.5·2.7 g^−1^·kg^−1^) (15), and performing a minimal aerobic routine only (30 minutes per week), during the cutting phase. This mild and infrequent aerobic exercise represents a minimal stimulus compared with the aerobic routines that are frequently performed by competitive bodybuilders (14). The subject of this study likely adopted this strategy in the attempt to minimize the loss of fat-free mass. Fat mass indeed was already extremely low in the bulking phase. Moreno et al. (25) assessed an Open category bodybuilder with a body mass of 131.4 kg and 12.2% of body fat in a single-visit assessment, and the lower fat mass observed in our subject (in both phases) may reflect differences in the nutritional and training strategies used by the 2 athletes. However, further direct comparisons with other athletes were not possible because previous studies involving Open category athletes reported the overall MT rather than the values of the individual muscles (25).

The analysis of muscle architecture in the 2 phases revealed a reduction in MT in all the considered sites, and the drop was particularly evident in muscles such as the medial gastrocnemius (−12.7%), the vastus lateralis (−12.2%), and the biceps femoris (−10.6%) in the lower body, and the trapezius (−28.4%), the pectoralis major (−5.8%), and the triceps brachii (−5.5%) in the upper body. This is consistent with the reduction in body mass and fat-free mass that was observed between the 2 phases. Contrary to what may be expected, the decrease in fat mass registered between the bulking and the cutting phases was not accompanied by a general reduction in muscle cEI. An increase in the cEI indeed was observed over time in almost all sites, suggesting that changes in muscle quality may be independent by the reductions of MT occurring during the cutting phase. The biceps femoris and the lateral gastrocnemius showed the most relevant increases of cEI in the lower body (+30.5% and +8.6%, respectively), while substantial changes were registered in the upper body, in the elbow flexors (+28.5%), the triceps brachii (+13.2%), and the upper trapezius (+12.5%). Previous studies associated EI to “muscle quality,” and the values registered in our subject are among the lowest reported in scientific literature (5,7,20). The extreme muscle hypertrophy of our subject and the minimal fat percentage may influence the amount of intramuscular fat (13). Intramuscular fat, together with muscle glycogen, are considered the most important factors determining EI and muscle quality (37). In addition, animal studies suggested that low levels of intramuscular fat, resulting in a low EI, were registered in muscles characterized by a high percentage of fast fibers (18). These relationship between the EI and the prevalent type of muscle fibers, however, to date, has not yet been sufficiently investigated in humans. Thus, the increase in cEI registered in the cutting phase, compared with the bulking phase, without increases of fat mass, may reflect a state of stress and catabolism of the muscle, potentially resulting in a substantial reduction of muscle glycogen. These findings also showed a muscle-specific response to the cutting phase in our athlete, suggesting that some muscles (e.g., upper trapezius, biceps femoris, and triceps brachii), may be more sensitive to energy restriction than others.

Because the subject competes in IFBB Pro League contests, in which the use of performance-enhancing drugs cannot be excluded, muscle adaptations to the different training strategies and dietary interventions may differ greatly by those achievable in natural athletes. Future studies indeed should investigate changes in muscle architecture and muscle quality in the different phases of preparation for a natural bodybuilding competition.

A potential limitation of this study is represented by the use of the equation of Evans et al. (11) to estimate body fat. Although this equation was developed for athletes, it was not specifically validated for elite bodybuilders. Thus, the percentages of body fat reported in this study may not be accurate. Therefore, the sum of the skinfolds and the single skinfolds were also reported in the article. In addition, 3-site equations are typically less accurate than more comprehensive protocols (e.g., ISAK 8-site) (19) especially when a single individual is measured and the differences in regional fat distribution are not averaged out across a sufficient sample of subjects to make summary statistics more representative. That may be particularly true in bodybuilders, in which 8-site assessments may also increase the ecological validity of the measurement, representing more accurately a parameter that directly affects competitive performance.

In conclusion, this is the first study showing that the energy restriction characterizing the cutting phase of a bodybuilding preparation may affect muscle quality and muscle mass, with different impacts on the different muscles. In addition, changes in cEI may indicate a suboptimal muscle condition in the cutting compared with the bulking phase of an extremely lean and muscular athlete.

**Practical Applications**

The assessment of body composition, together with the evaluation of the muscle architecture (muscle thickness and corrected echo-intensity), may help athletes and coaches in monitoring adaptations and optimizing strategies throughout a bodybuilding preparation. In particular, the combination between MT and EI values should be assessed throughout both preparation phases to better understand the individual responses of key muscle groups to the different nutritional and training interventions adopted.
